# One step synthesis of high-efficiency AgBr–Br–g-C_3_N_4_ composite catalysts for photocatalytic H_2_O_2_ production *via* two channel pathway

**DOI:** 10.1039/c8ra07749e

**Published:** 2018-11-01

**Authors:** Yunke Wang, Shaozheng Hu, Qiang Li, Guizhou Gu, Yanfeng Zhao, Hongyu Liang, Wei Li

**Affiliations:** College of Chemistry, Chemical Engineering, and Environmental Engineering, Liaoning Shihua University Fushun 113001 China weililnpu@163.com

## Abstract

In this work, a two-component modified AgBr–Br–g-C_3_N_4_ composite catalyst with outstanding photocatalytic H_2_O_2_ production ability is synthesized. XRD, UV-Vis, N_2_ adsorption, TEM, XPS, EPR and PL were used to characterize the obtained catalysts. The as-prepared AgBr–Br–g-C_3_N_4_ composite catalyst shows the highest H_2_O_2_ equilibrium concentration of 3.9 mmol L^−1^, which is 7.8 and 19.5 times higher than that of GCN and AgBr. A “two channel pathway” is proposed for this reaction system which causes the remarkably promoted H_2_O_2_ production ability. In addition, compared with another two-component modified catalyst, Ag–AgBr–g-C_3_N_4_, AgBr–Br–g-C_3_N_4_ composite catalyst displays the higher photocatalytic H_2_O_2_ production ability and stability.

## Abstract 2

In this work, a two-component modified AgBr–Br–g-C_3_N_4_ composite catalyst with outstanding photocatalytic H_2_O_2_ production ability is synthesized.

## Graphical abstract

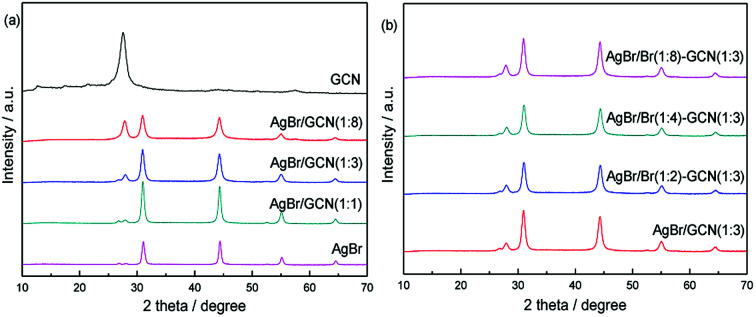

## Introduction

Hydrogen peroxide (H_2_O_2_) is a highly efficient and green oxidant because it has a high content of active oxygen (47% w/w) and results in only H_2_O as a by-product.^1,2^ Industrially, H_2_O_2_ is produced by the anthraquinone method, in which energy consumption is high because of the multistep hydrogenation and oxidation reactions. Thus this method is unsuitable for the current new concept of being “green, energy saving, and environmentally friendly” in the chemical industry. Recently, the direct synthesis of H_2_O_2_ from H_2_ and O_2_ gases has been widely studied using noble metals as catalysts.^3,4^ It is considered to be an alternative and green chemical process. However, this method presents a potential explosion risk from H_2_/O_2_ mixtures. In contrast, photocatalytic H_2_O_2_ production requires only water, oxygen and visible light through two-electron reduction from the conduction band (reaction (1)). However, the H_2_O_2_ can be decomposed by reduction with e^−^, which has caused the H_2_O_2_ production rate of this photocatalytic reduction method to be unsatisfactory to date (reaction (2)).

1 O_2_ + 2H^+^ + 2e^−^ → H_2_O_2_

2 H_2_O_2_ + e^−^ → ·OH + OH^−^

Graphite phase carbon nitride (g-C_3_N_4_), as the darling of the catalytic community in recent years, has special physical and chemical properties, excellent chemical stability and adjustable electronic structure.^5,6^ Since its conduction band potential (−1.3 V) is more negative than the reduction potential of O_2_/H_2_O_2_ (0.695 V), g-C_3_N_4_ could reduce O_2_ to H_2_O_2_ under visible light thermodynamically.^7^ However, g-C_3_N_4_ also suffers from many disadvantages, such as the low visible-light utilization efficiency, high recombination rate and small BET surface area, which limit its practical application.

AgX (X = Cl, Br, I) is a kind of common photocatalyst. They have the moderate energy level which is very appropriate to combine with g-C_3_N_4_ for building heterojunction to resolve these disadvantages mentioned above.^8–14^ Feng *et al.* prepared g-C_3_N_4_/AgBr nanocomposite photocatalyst *via* a protonation pretreatment method.^8^ They found the efficient combination of g-C_3_N_4_ and AgBr leads to *Z*-scheme charge transfer in the composite system, and the photocatalytic activity is therefore enhanced significantly. Liu *et al.* prepared AgI@g-C_3_N_4_ hybrid core@shell structure by ultrasonication/chemisorption method.^9^ They suggested that the improved photocatalytic performance is due to synergistic effects at the interface of AgI and g-C_3_N_4_ which can effectively accelerate the charge separation and reinforce the photostability of hybrid composite. In addition of building heterojunction with silver halide, halogen doping and Ag loading are also efficient method to promote the visible-light utilization efficiency and separation rate of electrons–holes. Lan *et al.* prepared Br doped g-C_3_N_4_ for photoredox water splitting *via* one-pot co-condensation of urea with NH_4_Br.^15^ The optimal sample shows more than two times higher H_2_ evolution rates than pure g-C_3_N_4_ under visible light irradiation, with high stability during the prolonged photocatalytic operation. Bu *et al.* prepared Ag loaded mesoporous g-C_3_N_4_ with high photoelectric conversion performance.^16^ They suggested that modifying mesoporous g-C_3_N_4_ with Ag increases the conductivity and lowers the energy barrier of the interface reactions, thus enhances the separation efficiency of photogenerated electron–hole pairs.

Besides, two-component modification, such as Ag–AgX, are also used to promote the catalytic performance of g-C_3_N_4_.^17,18^ Wang *et al.* prepared a high-efficiency g-C_3_N_4_/Ag/AgCl plasmonic photocatalyst *via* a facile solvothermal method.^17^ The surface plasmon resonance effect of the Ag nanoparticles, the polarization field of AgCl and the g-C_3_N_4_/Ag/AgCl heterojunction all result in the improved photocatalytic degradation performance. Chen *et al.* prepared plasmonic photocatalyst Ag/AgBr/g-C_3_N_4_ by *in situ* ionic-liquid-assisted synthesis.^18^ The enhanced photocatalytic activity is assigned to the extended light-absorption range and efficient charge separation caused by the surface plasmon resonance effect of Ag^0^ and the well-matched overlapping band-structure between Ag/AgBr and g-C_3_N_4_. Interestingly, till now, few literature concerning silver halide–halogen–g-C_3_N_4_ catalyst is reported. F and I have been applied to dope g-C_3_N_4_. It was found that F dopant give much less promotional effect on the optical absorption of g-C_3_N_4_ than I.^19^ This is because the valence electrons in I atom with much less electronegativity are more delocalized to interact with the π electron system of g-C_3_N_4_. Such an extended conjugation system gives rise to the red-shift of the optical absorption of g-C_3_N_4_. However, the overlarge size of I atom is thermodynamically and geometrically difficult to dope into g-C_3_N_4_ to form stable structure. To this end, Br in the middle of F and I is a recommended choice to modify g-C_3_N_4_. In this work, AgBr–Br–g-C_3_N_4_ composite catalyst is prepared *via* one-pot synthesis. The as-prepared AgBr–Br–g-C_3_N_4_ composite catalyst is applied for photocatalytic H_2_O_2_ production under visible light irradiation. The effects of modification on the structural property, optical property, and photocatalytic performance of catalysts are discussed in detail. The possible reaction mechanism is proposed.

## Experimental

### Preparation and characterization

4 g of dicyandiamide and desired amount of AgNO_3_ were dissolved into 40 mL deionized water under stirring to obtain solution A. Then, NH_4_Br solution was added dropwise into solution A (molar ratio Ag/Br = 1). The obtained suspension was stirred for 2 h, and heated to 80 °C to remove the water. The solid was dried at 80 °C and followed by milling and annealing at 550 °C for 2 h (at a rate of 5 °C min^−1^). The obtained catalyst was denoted as AgBr/GCN(*x*), where *x* stands for the mass ratio of AgBr/dicyandiamide. Neat AgBr was prepared according to the method mentioned above in the absence of dicyandiamide. When excess NH_4_Br was added into solution A and followed the same procedure mentioned above, the prepared catalyst was denoted as AgBr/Br(*y*)–GCN(*x*), where *y* stands for the molar ratio of Ag/Br.

The XRD patterns of the samples were recorded on a Rigaku D/max-2400 instrument using Cu-Kα radiation (*λ* = 1.54 Å). UV-Vis spectroscopy was carried out on a UV-Vis spectrophotometer (JASCO V-550) using BaSO_4_ as the reflectance sample. TEM images were taken on a Philips Tecnai G220 model microscope. Nitrogen adsorption was measured at −196 °C on a Micromeritics 2010 analyzer. The BET surface area (*S*_BET_) was calculated based on the adsorption isotherm. Elemental analysis was performed using a vario EL cube from Elementar Analysensysteme GmbH. ICP was performed on a Perkin-Elmer Optima 3300DV apparatus. The XPS results were obtained on a Thermo Escalab 250 XPS system. Al Kα radiation was used as the excitation source. The electron paramagnetic resonance (EPR) was determined with a Bruker ESR 300E, using the radical scavenger dimethyl pyridine N-oxide (DMPO). The photoluminescence (PL) spectra were measured with a fluorospectrophotometer (FP-6300) using Xe lamp as the excitation source.

### Photocatalytic reaction

The photocatalytic H_2_O_2_ production ability of the samples was evaluated by the reduction of molecular oxygen. For these experiments, 0.2 g of photocatalyst was added to 200 mL of deionized water. The suspension was dispersed using an ultrasonicator for 10 min. During the photoreaction under visible light irradiation, the suspension was exposed to a 250 W high-pressure sodium lamp with main emission from 400 to 800 nm, and O_2_ was bubbled at 80 mL min^−1^ through the solution. The UV light portion of the sodium lamp was filtered by a 0.5 M NaNO_2_ solution. All runs were conducted at ambient pressure and 30 °C. At given time intervals, 5 mL aliquots of the suspension were collected and immediately centrifuged to separate the liquid samples from the solid catalyst. The H_2_O_2_ concentration was analyzed by the normal iodometric method.^20,21^

## Results and discussion

**Fig. 1:**
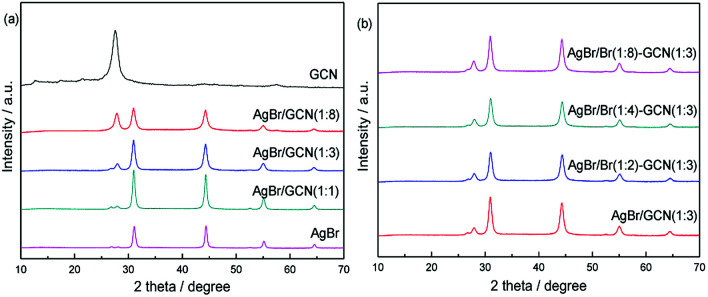
XRD patterns of as-prepared catalysts.

Fig. 1 shows the XRD patterns of GCN, AgBr and as-prepared composite catalysts. The characteristic peak of GCN around 27.5° could be clearly identified, which is attributed to the typical (0 0 2) interlayer-stacking peak corresponds to an interlayer distance of *d* = 0.33 nm. The peak at 13.1° represents in-plane structural packing motif with a *d* value of 0.675 nm.^22^ For AgBr, the sample shows several diffraction peaks at 31.0°, 44.4°, 55.1° and 64.6°, which are assigned to the (2 0 0), (2 2 0), (2 2 2) and (4 0 0) planes of AgBr crystal (JCPDS file: 6-438).^8,18^ In the case of as-prepared composite catalysts, the peak position does not shift but the intensity for AgBr (GCN) decreases (increases) gradually with increasing the amount of GCN, as shown in Fig. 1a. It is shown in Fig. 1b that, when excess Br was added, the peak intensity and position for both GCN and AgBr are not changed. In addition, the element analysis and ICP results indicate that, the AgBr concentrations in AgBr/GCN(1 : 8), AgBr/GCN(1 : 3) and AgBr/GCN(1 : 1) are 26 wt%, 42 wt% and 69 wt%, respectively. For AgBr/Br(1 : 2)–GCN(1 : 3), AgBr/Br(1 : 4)–GCN(1 : 3) and AgBr/Br(1 : 8)–GCN(1 : 3), the doping Br concentrations are 0.25 wt%, 0.44 wt% and 0.72 wt%, respectively.

**Fig. 2:**
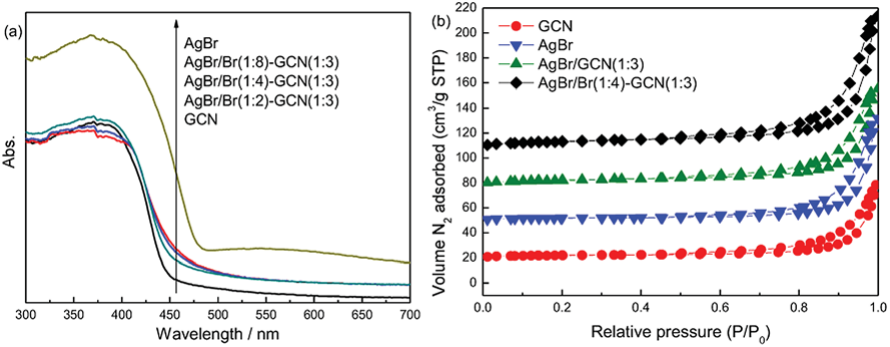
UV-Vis (a) and N_2_ adsorption–desorption isotherms (b) of as-prepared catalysts.

Fig. 2a displays the UV-Vis spectra of as-prepared catalysts. GCN shows the typical absorption spectrum of graphitic carbon nitride semiconductor with an absorption boundary at approximately 450 nm. For AgBr, the absorption in the whole region is stronger than that of GCN. Its absorption boundary is around 490 nm. In the case of as-prepared composite catalysts, compared with GCN, their absorption boundaries are shifted slightly to 460–470 nm. The higher Br/Ag molar ratio, the larger shift of absorption boundary. In addition, because of the presence of AgBr, the composite catalysts display the increased absorption in the whole visible light region. The band gaps of samples were calculated according to the method of Oregan and Gratzel.^23^ The result shows that the band gap for GCN, AgBr/Br(1 : 2)–GCN(1 : 3), AgBr/Br(1 : 4)–GCN(1 : 3), AgBr/Br(1 : 8)–GCN(1 : 3) and AgBr is 2.75, 2.69, 2.65, 2.63 and 2.53 eV, respectively. This indicates that Br doping influence the energy band position of as-prepared catalysts. Fig. 2b displays the N_2_ adsorption–desorption isotherms of as-prepared catalysts. All the catalysts exhibit type IV isotherm. The specific surface area (*S*_BET_) of GCN, AgBr and AgBr/GCN(1 : 3), calculated by the BET method, are 9.5, 13.2 and 10.9 m^2^ g^−1^, respectively. In the case of AgBr/Br(1 : 4)–GCN(1 : 3), its *S*_BET_ is 11.4 m^2^ g^−1^, very close to that of AgBr/GCN(1 : 3). This hints Br doping does not influence the *S*_BET_ of catalyst.

**Fig. 3:**
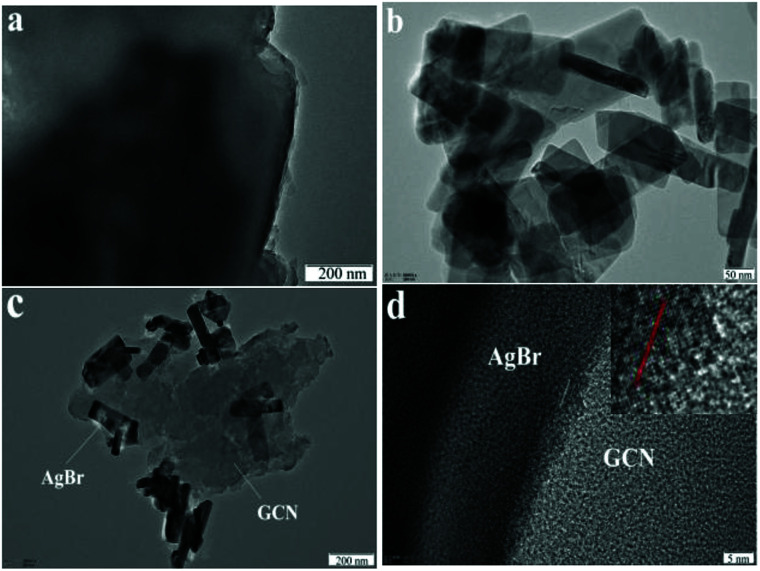
TEM images of GCN (a), AgBr (b) and AgBr/Br(1 : 4)–GCN(1 : 3) (c and d).

The morphologies of the representative samples were examined by TEM analysis. As shown in Fig. 3a, GCN shows sheet-like structure with no regular morphology. The morphology for AgBr is nanorod, as shown in Fig. 3b. In the case of AgBr/Br(1 : 4)–GCN(1 : 3) (Fig. 3c), both plate-like structural GCN and AgBr nanorod are observed, confirming the presence of both GCN and AgBr. The two-dimensional ordering of GCN is very weak and hard to find the lattice fringe in HRTEM image (Fig. 3d). This is consistent with previous work.^18^ However, the clear lattice fringe is observed for AgBr, very close to the (400) crystal face with the *d* = 0.143 nm (Fig. 3d). This tight coupling is favorable for the charge transfer between GCN and AgBr and promotes the separation rate of electron–hole pairs.

**Fig. 4:**
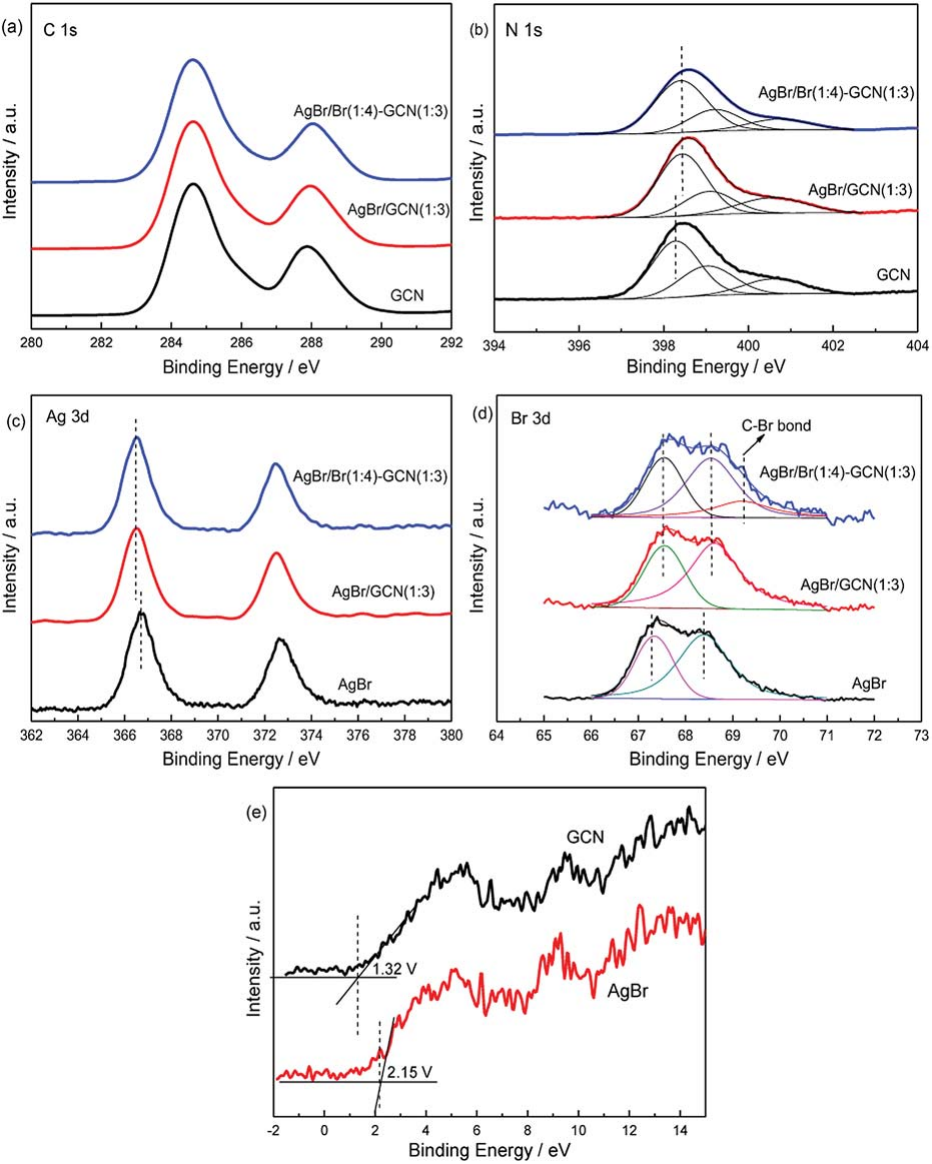
XPS of as-prepared catalysts in the C 1s (a), N 1s (b), Ag 3d (c), Br 3d (d) and VB XPS (e) regions.

XPS spectra are used to investigate the structure of the as-prepared catalysts. In Fig. 4a, the spectra of three catalysts in C 1s region can be fitted with two contributions which located at 284.6 and 288.6 eV. The sharp peak around 284.6 eV is attributed to the pure graphitic species in the CN matrix. The peak with binding energy of 288.6 eV indicates the presence of sp^2^ C atoms bonded to aliphatic amine (–NH_2_ or –NH–) in the aromatic rings.^24–26^ For N 1s region (Fig. 4b), three contributions located at 398.3, 399 and 400.5 eV were assigned to the sp^2^ hybridized aromatic nitrogen atoms bonded to carbon atoms (C–N=C), tertiary nitrogen N–(C)_3_ groups linking structural motif or amino groups carrying hydrogen ((C)_2_–N–H) in connection with structural defects and incomplete condensation, and nitrogen atoms bonded three carbon atoms in the aromatic cycles.^27,28^ For AgBr/GCN(1 : 3) and AgBr/Br(1 : 4)–GCN(1 : 3), the obvious shifts to higher binding energies are observed in N 1s region compared with that of neat GCN. This is probably due to the change of chemical environment after coupling with AgBr.

In Fig. 4c, AgBr displays Ag 3d spectrum with two peaks at 366.8 eV and 372.8 eV, corresponding to the binding energies of Ag 3d_5/2_ and Ag 3d_3/2_ of Ag^+^ in AgBr, respectively.^29^ In Br 3d region (Fig. 4d), the peaks at ∼67.5 and 68.3 eV for AgBr are assigned to the Br 3d_5/2_ and Br 3d_3/2_ of Br^−^ state.^30^ For AgBr/GCN(1 : 3), the binding energies shift obviously in both Ag 3d and Br 3d regions compared with neat AgBr, which confirms the strong interaction between AgBr and GCN. AgBr/Br(1 : 4)–GCN(1 : 3) displays the same peak position with AgBr/GCN(1 : 3) in both Ag 3d and Br 3d regions. Moreover, a new peak at 69.4 eV is observed for AgBr/Br(1 : 4)–GCN(1 : 3). This binding energy should be assigned to the C–Br bond, which confirming that Br is doped into g-C_3_N_4_ lattice.^31^ The VB XPS spectra were employed to determine the electronic structure (Fig. 4e). It is obvious to see that the VB potentials for GCN and AgBr locate at +1.32 and +2.15 V. They are very close to the previous results.^5,18^ Combined with the UV-Vis results, the energy position of CB for GCN and AgBr locate at −1.43 and −0.38 V respectively. Obviously, the band structures of the two components are well-matched with each other. This facilitates the formation of heterojunction for charge transfer.

**Fig. 5:**
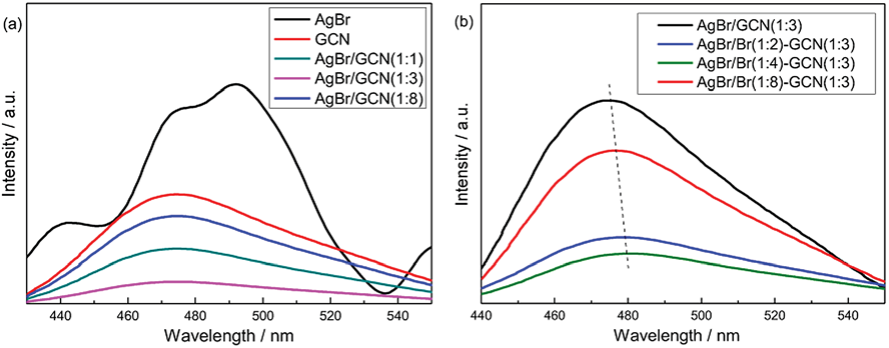
PL spectra of as-prepared catalysts.

Fig. 5 shows the PL spectra of as-prepared catalysts under air atmosphere using excitation at 255 nm. For GCN (Fig. 5a), broad PL band around 460 nm is observed with the energy of light approximately equal to the band gap of g-C_3_N_4_. AgBr exhibits several emission peaks which intensities are higher than that of GCN. In the case of as-prepared heterojunction catalysts, the PL spectra show the similar shape to that of GCN, whereas the intensities are obviously decreased. AgBr/GCN(1 : 3) shows the lowest PL intensity, hinting its most effective separation rate of electrons and holes. This is reasonable because, with this GCN/AgBr mass ratio, GCN and AgBr have the approximate *S*_BET_ (9.5 and 13.2 m^2^ g^−1^ for GCN and AgBr, the yield for GCN is approximately 50 wt%). They can contact with each other as much as possible, leading to the formation of the maximum area of the heterojunction.

In Fig. 5b, it is shown that, compared with AgBr/GCN(1 : 3), the PL intensity is sharply decreased for AgBr/Br(1 : 2)–GCN(1 : 3) and AgBr/Br(1 : 4)–GCN(1 : 3), accompanying with the obvious red-shift. This is probably due to that the doping Br atoms can induce strong spin–orbit interaction in GCN and result in the conversion of singlet-excited GCN into triplet-excited GCN. For AgBr/Br(1 : 8)–GCN(1 : 3), its PL intensity is much higher than that of AgBr/Br(1 : 2)–GCN(1 : 3) and AgBr/Br(1 : 4)–GCN(1 : 3), but still lower than AgBr/GCN(1 : 3). Such PL quenching is possible due to the non-radiative transition.

**Fig. 6:**
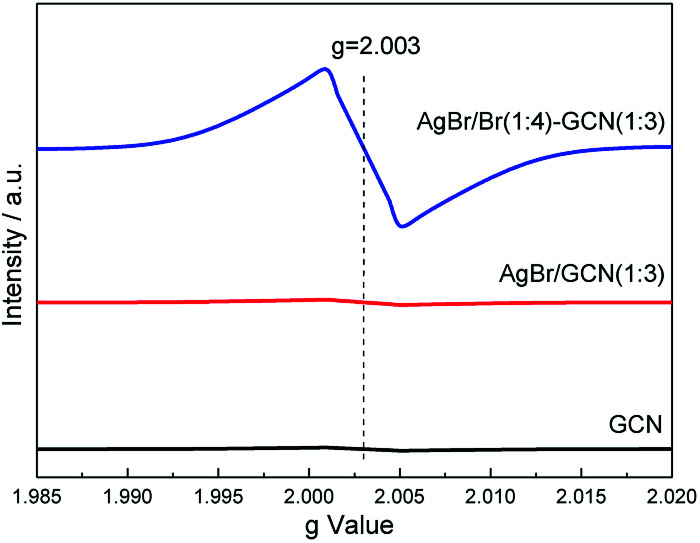
EPR spectra of as-prepared GCN, AgBr/GCN(1 : 3) and AgBr/Br(1 : 4)–GCN(1 : 3).

Room temperature electron paramagnetic resonance (EPR) was used to investigate the electronic property of the as-prepared catalysts. As shown in Fig. 6, GCN shows almost no EPR signal. After coupling with AgBr, the sample also displays no EPR signal for AgBr/GCN(1 : 3). However, AgBr/Br(1 : 4)–GCN(1 : 3) shows one single Lorentzian line centering at a *g* = 2.003, being originated from unpaired electrons on π-conjugated g-C_3_N_4_ aromatic rings after Br doping. This is probably due to that the delocalization of the valance electron of Br in the g-C_3_N_4_ conjugation system can widen the band distribution, which improves the charge migration.^15^ This is consistent with the PL result.

**Fig. 7:**
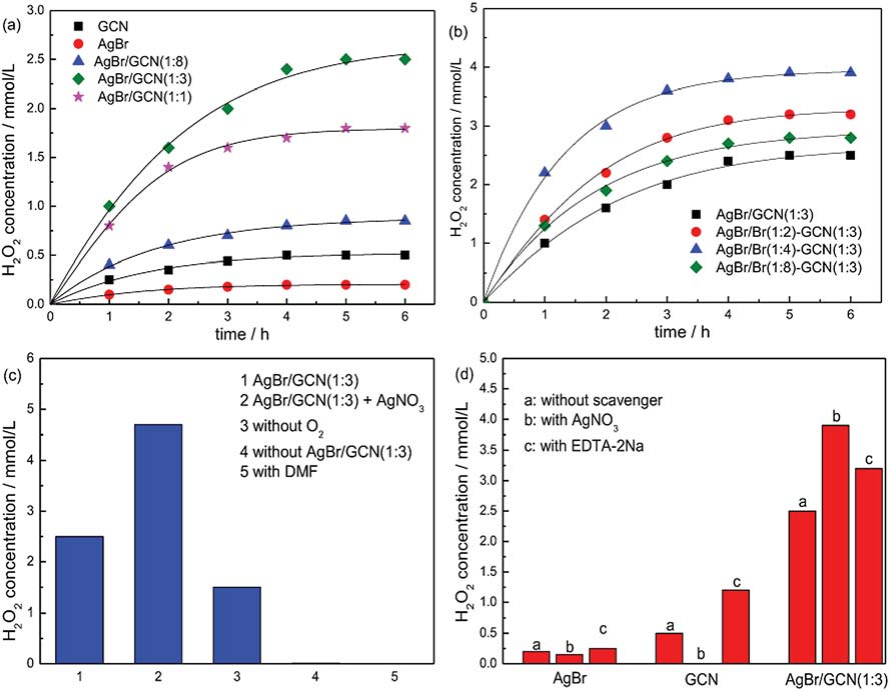
The H_2_O_2_ production ability over as-prepared catalysts (a and b), the H_2_O_2_ production ability of AgBr/GCN(1 : 3) under different reaction conditions (c) and the influence of various scavengers on the H_2_O_2_ production ability over as-prepared catalysts (d).

Fig. 7 displays the photocatalytic H_2_O_2_ production ability over as-prepared catalysts. It is clearly seen that the H_2_O_2_ concentration of as-prepared catalyst increases with time for about 5 h when it reaches a constant level. This level corresponds to the steady-state where the rate of H_2_O_2_ production is equal to the rate of decomposition.^32^ In Fig. 7a, GCN and AgBr display the H_2_O_2_ concentration of 0.5 and 0.2 mmol L^−1^. In the case of as-prepared heterojunction catalyst, the H_2_O_2_ production ability promotes obviously. After Br doping, the photocatalytic performance of as-prepared samples further increase, as shown in Fig. 7b. This is due to that the introduction of heteroatoms into the π-conjugated g-C_3_N_4_ can accelerate the charge carriers transfer rate and thus restrain the recombination of electron and hole. AgBr/Br(1 : 4)–GCN(1 : 3) shows the highest H_2_O_2_ equilibrium concentration of 3.9 mmol L^−1^, which is 7.8 and 19.5 times higher than that of GCN and AgBr. The excess Br doping causes the decreased H_2_O_2_ production ability of AgBr/Br(1 : 8)–GCN(1 : 3). This is probably due to that the excess doping Br acts as recombination sites to accelerate the recombination of electrons and holes, which is consistent with PL result.

Fig. 7c shows the H_2_O_2_ production ability of AgBr/GCN(1 : 3) under different reaction conditions. The addition of AgNO_3_ (10 mmol L^−1^) as an electron scavenger should suppress the H_2_O_2_ production ability. However, interestingly, the H_2_O_2_ production ability of AgBr/GCN(1 : 3) increases to 4.7 mmol L^−1^ when AgNO_3_ is added. Still 1.5 mmol L^−1^ H_2_O_2_ is obtained in the absence of O_2_. These results indicate that H_2_O_2_ is not only produced by O_2_ reduction over AgBr/GCN(1 : 3). The detailed explanation is given below. The H_2_O_2_ concentration is trivial in the absence of photocatalyst, indicating that H_2_O_2_ is produced *via* a photocatalytic process. No H_2_O_2_ is generated when using dimethylformamide (DMF, aprotic solvent) instead of water, confirming that H_2_O is necessary as the proton source for H_2_O_2_ production.

3 h^+^ + OH^−^ → ·OH

4 ·OH + ·OH → H_2_O_2_

Besides the O_2_ reduction to form H_2_O_2_, another channel to produce H_2_O_2_ is reported by Dong *et al.*^33^ It is reported that photogenerated holes can oxidize OH^−^ to ·OH thermodynamically, as shown in reaction (3). Two ·OH can form H_2_O_2_ though combination with each other, as shown in reaction (4). In order to investigate the reaction mechanism of as-prepared heterojunction catalyst, the influence of various scavengers on the H_2_O_2_ production ability is carried out and shown in Fig. 7d. AgNO_3_ and EDTA-2Na are used as the electrons (e^−^) and hole (h^+^) scavenger, respectively.^34^ When AgNO_3_ is added to trap the electrons, H_2_O_2_ is still formed with the concentration of 0.15 mmol L^−1^ over AgBr. It is known that the redox potential for ·OH/OH^−^ is +1.99 V.^35^ Whereas the VB of AgBr is +2.15 V. The VB holes in AgBr is positive enough to generate ·OH. Therefore, H_2_O_2_ should be produced by reaction (4). When EDTA-2Na is added to trap the holes, although the utilization rate of electrons is promoted, the H_2_O_2_ equilibrium concentration is only increased to 0.45 mmol L^−1^ over AgBr. This is probably due to the poor reduction ability of CB electrons over AgBr. In the case of GCN, the CB and VB positions are −1.43 V and +1.32 V, respectively. Thus, when AgNO_3_ is added, no H_2_O_2_ is produced due to that the VB holes in g-C_3_N_4_ are not positive enough to generate ·OH. Without any doubt, the H_2_O_2_ production ability of g-C_3_N_4_ is obviously promoted by adding EDTA-2Na to trap the holes.

**Fig. 8:**
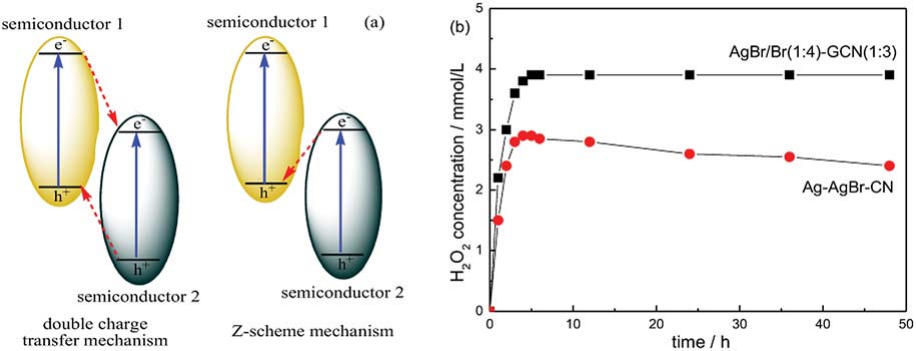
The sketch map of double charge transfer mechanism and *Z*-scheme mechanism over heterojunction catalyst (a) and the photocatalytic H_2_O_2_ production ability over AgBr/Br(1 : 4)–GCN(1 : 3) and Ag–AgBr–CN (b).

In general, there are two typical working mechanisms for heterojunction catalyst, double charge transfer mechanism and *Z*-scheme mechanism, as shown in Fig. 8a.^36–38^ If AgBr/GCN(1 : 3) catalyst follows the double charge transfer mechanism, holes should be transferred to the VB of GCN. When CB electrons is trapped by AgNO_3_, VB holes in GCN are not positive enough to generate ·OH. Thus H_2_O_2_ should be not produced. However, the H_2_O_2_ with the concentration of 3.9 mmol L^−1^ is formed over AgBr/GCN(1 : 3) when AgNO_3_ is added. Similarly, if AgBr/GCN(1 : 3) follows the double charge transfer mechanism, the electrons should be transferred to the CB of AgBr. The H_2_O_2_ production ability should be low by adding EDTA-2Na to trap the holes because of the poor reduction ability of CB electrons over AgBr. Whereas, the H_2_O_2_ equilibrium concentration over AgBr/GCN(1 : 3) is promoted to 3.2 mmol L^−1^ when EDTA-2Na is added. Based on the above results, it is deduced that not double charge transfer mechanism but *Z*-scheme mechanism with “two channel pathway” is proposed. Under visible light irradiation, the photogenerated electron–hole pairs are formed in both components. The electrons in the CB of AgBr combine with the holes in the VB of GCN at the interface of the heterojunction. Therefore, the holes tend to stay in the VB of AgBr and the electrons accumulate in the CB of GCN, leading to the enhanced separation rate of electron–hole pairs. The CB electrons in GCN can reduce O_2_ to form H_2_O_2_, as well as the VB holes in AgBr can oxidize OH^−^ to form ·OH, which subsequently react with each other to form H_2_O_2_. Such “two channel pathway” causes the remarkably promoted H_2_O_2_ production ability. When AgNO_3_ (or EDTA-2Na) is added to trap the electrons (or holes), the separation efficiency of catalyst is promoted, leading to the enhanced H_2_O_2_ production ability (Fig. 7d).

In order to prove the advance of this AgBr–Br–GCN heterojunction catalyst, Ag–AgBr–CN was prepared according to the previous work.^18^Fig. 8b compares the photocatalytic H_2_O_2_ production ability of AgBr/Br(1 : 4)–GCN(1 : 3) and Ag–AgBr–CN. Ag–AgBr–CN shows the H_2_O_2_ concentration of 2.9 mmol L^−1^, much lower than that of AgBr/Br(1 : 4)–GCN(1 : 3). In addition, the H_2_O_2_ production ability for AgBr/Br(1 : 4)–GCN(1 : 3) keeps stable after 48 h reaction. Whereas, the activity for Ag–AgBr–CN decreases obviously with increasing the reaction time, hinting its poor photocatalytic stability. The element analysis and ICP results indicate that the content of each element in the AgBr/Br(1 : 4)–GCN(1 : 3) remains almost unchanged before and after the reaction. However, for Ag–AgBr–CN, the Ag content decreases obviously (Br content does not change). This indicates the metal silver is not sturdy on the catalyst surface, which is probably lost during the reaction.

## Conclusions

In this work, a two-component modified AgBr–Br–g-C_3_N_4_ composite catalyst with outstanding photocatalytic H_2_O_2_ production ability is synthesized. Modification with Br and AgBr does not change the structural property of g-C_3_N_4_ but decreases the band gap and increases the visible light absorption. The formation of heterojunction with AgBr and introduction of heteroatoms Br into the π-conjugated g-C_3_N_4_ can accelerate the charge carriers transfer rate and thus restrain the recombination of electron and hole. AgBr/Br(1 : 4)–GCN(1 : 3) shows the highest H_2_O_2_ equilibrium concentration of 3.9 mmol L^−1^, which is 7.8 and 19.5 times higher than that of GCN and AgBr. According to “*Z*-scheme” mechanism, not only the CB electrons of GCN reduce O_2_ to form H_2_O_2_, but the VB holes in AgBr can oxidize OH^−^ to form ·OH, which subsequently react with each other to form H_2_O_2_. Such “two channel pathway” causes the remarkably promoted H_2_O_2_ production ability. In addition, compared with another two-component modified catalyst, Ag–AgBr–g-C_3_N_4_, AgBr/Br(1 : 4)–GCN(1 : 3) displays the higher photocatalytic H_2_O_2_ production ability and stability.

## Conflicts of interest

There are no conflicts to declare.

## Supplementary Material
